# Fluid management in ARDS: an evaluation of current practice and the association between early diuretic use and hospital mortality

**DOI:** 10.1186/s40560-020-00496-7

**Published:** 2020-10-12

**Authors:** Kevin P. Seitz, Ellen S. Caldwell, Catherine L. Hough

**Affiliations:** 1 Division of Pulmonary, Allergy, and Critical Care Medicine, Vanderbilt University, Nashville, TN USA; 2 Vanderbilt University Medical Center, T1218 MCN, 1161 21st Avenue, Nashville, TN 37232 USA; 3 Division of Pulmonary and Critical Care Medicine, University of Washington, Seattle, WA USA; 4 Division of Pulmonary and Critical Care Medicine, Oregon Health and Science University, Portland, OR USA

**Keywords:** Acute respiratory distress syndrome, Diuretics, Fluid therapy, Critical care

## Abstract

**Background:**

Acute respiratory distress syndrome (ARDS) and volume overload are associated with increased hospital mortality. Evidence supports conservative fluid management in ARDS, but whether current practice reflects the implementation of that evidence has not been described. This study reports the variability in contemporary fluid management for ICU patients with ARDS. We compared routine care to trial protocols and analyzed whether more conservative management with diuretic medications in contemporary, usual care is associated with outcomes.

**Methods:**

We performed a retrospective cohort study in nine ICUs at two academic hospitals during 2016 and 2017. We included 234 adult patients with ARDS in an ICU at least 3 days after meeting moderate-severe ARDS criteria (PaO_2_:FIO_2_ ≤ 150). The primary exposure was any diuretic use in 48 to 72 h after meeting ARDS criteria. The primary outcome was hospital mortality. Unadjusted statistical analyses and multivariable logistic regression were used.

**Results:**

In 48–72 h after meeting ARDS criteria, 116 patients (50%) received a diuretic. In-hospital mortality was lower in the group that received diuretics than in the group that did not (14% vs 25%; *p* = 0.025). At ARDS onset, both groups had similar Sequential Organ Failure Assessment scores and ICU fluid balances. During the first 48 h after ARDS, the diuretic group received less crystalloid fluid than the no diuretic group (median [inter-quartile range]: 1.2 L [0.2–2.8] vs 2.4 L [1.2-5.0]; *p* < 0.001), but both groups received more fluid from medications and nutrition than from crystalloid. At 48 h, the prevalence of volume overload (ICU fluid balance >10% of body weight) in each group was 16% and 25%(*p* = 0.09), respectively. During 48–72 h after ARDS, the overall prevalence of shock was 44% and similar across both groups. Central venous pressure was recorded in only 18% of patients. Adjusting for confounders, early diuretic use was independently associated with lower hospital mortality (AOR 0.46, 95%CI [0.22, 0.96]).

**Conclusions:**

In this sample of ARDS patients, volume overload was common, and early diuretic use was independently associated with lower hospital mortality. These findings support the importance of fluid management in ARDS and suggest opportunities for further study and implementation of conservative fluid strategies into usual care.

## Background

Acute respiratory distress syndrome (ARDS) is a life-threatening condition marked by hypoxemic respiratory failure and non-cardiogenic pulmonary edema [1, 2]. Despite decades of focused research, ARDS remains common in critically ill patients and is associated with high mortality [3, 4]. In the management of patients with ARDS, the accumulation of a positive fluid balance has been associated with increased duration of mechanical ventilation and mortality, but fluid balance is a potentially modifiable risk factor for these poor outcomes [5–10].

With the Fluids and Catheters Treatment Trial (FACTT) in 2007, Wiedemann and collaborators demonstrated that a conservative fluid management strategy for ARDS reduces net fluid balance and improves outcomes in oxygenation and ventilator-free days [11]. The trial used a protocol guided by the central venous pressure (CVP) or pulmonary artery occlusion pressure (PAOP) to increase the use of diuretic medication and decrease the use of intravenous crystalloid. The protocol instructions established probable best practices for fluid management in ARDS [12–15]. These findings were supported further by a subsequent, less strict protocol (FACTT Lite) that also was associated with improved outcomes [16].

Despite the substantial body of evidence that positive fluid balance or volume overload could be harmful and that conservative fluid management may confer a benefit, few studies have characterized fluid balance in ARDS populations outside of clinical trials [8, 17]. It is not known whether conservative strategies, like early administration of diuretics, are used routinely in current practice for ARDS patients or whether they are associated with better outcomes when implemented in usual care.

This study describes the variability in fluid management among patients with moderate-severe ARDS in the intensive care unit (ICU). Specifically, we compare outcomes in patients who do and do not receive diuretic medications 48 to 72 h after meeting ARDS criteria, adjusting for clinical factors like severity of illness. We obtained empiric data about fluid balance and diuretic use in ARDS, and we hypothesized that early diuretic use would be independently associated with hospital mortality.

## Methods

### Study design

We conducted a retrospective cohort study of patients admitted to nine ICUs at two academic hospitals in Seattle, Washington from October 2016 to March 2017. We included all patients that had acute respiratory failure requiring intubation and mechanical ventilation in the ICU with moderate-severe ARDS as defined by a PaO_2_:FiO_2_ ratio ≤ 150 with bilateral opacities on chest imaging not fully explained by cardiac failure, lung collapse, nodules, or effusions [2]. Patients with chronic tracheostomies or admissions only for dialysis were excluded.

Patients were included from all ICUs in the two participating hospitals. Patients in these units are cared for by teaching services led by attending physicians with trainees. Daily rounds are practiced in all units with variability in processes of care like daily weights, and no protocols are in use for ARDS fluid management.

To capture the diversity of patients with ARDS as defined by evidence-based criteria, we excluded only those who first met ARDS criteria at an outside hospital and those who required renal replacement therapy, died, or were discharged from the ICU prior to 72 h after meeting ARDS criteria. Using the Berlin definition for ARDS, we did not exclude patients with a diagnosis of heart failure unless they also lacked an ARDS risk factor.

Data were abstracted from the electronic medical record by both automated methods and manual chart review for every patient. The institutional review board at the University of Washington reviewed and approved this study, waiving the need for informed consent.

### Primary variables

The primary outcome of interest was hospital mortality. Secondary outcomes were duration of mechanical ventilation and disposition at hospital discharge. Our primary exposure of interest was early diuretic use, defined as any diuretic use 48 to 72 h after meeting ARDS criteria. This time interval corresponds approximately to the first day of protocolized management in the FACTT trial, where enrollment was permitted up to 48 h after acute lung injury [11]. The secondary exposure of conservative fluid management was defined as the restrictive use of intravenous crystalloid fluids with less than 500 mL in that same 24-h period.

### Covariates

We collected patient demographics and clinical characteristics as covariates. Demographics included gender, race, age, and admission body weight. Clinical characteristics present at ARDS included chronic comorbidities, ARDS risk factors like trauma or sepsis as documented, and ICU admission directly from the operating room, ICU fluid balance before ARDS, and the Sequential Organ Failure Assessment (SOFA) score at the onset of ARDS [18].

The following clinical factors at 48 to 72 h after ARDS were defined a priori and evaluated as potential confounders because they were present at the time of diuretic management decisions and are known to be associated with mortality: shock, acute kidney injury (AKI), and persistently severe ARDS. Shock was defined as vasopressor use or mean arterial pressure <60 mmHg for two measurements. AKI was defined serum creatinine ≥2 mg/dL or oliguria (urine output <500 mL in 24 h) using criteria for renal failure established by the SOFA score for comparison with future studies. Persistently severe ARDS was defined by PaO_2_:FiO_2_ ≤ 150. Relevant electrolyte derangements were defined as any serum potassium <3 mEq/L, sodium >150 mEq/L, or bicarbonate >40 mEq/L.

Volume overload was defined by an ICU fluid balance of more than 10% of the hospital admission weight, as established by previous studies [19–22]. Daily fluid intake was characterized as crystalloid, free water, medication fluid, nutrition, and transfusions. Crystalloid includes any intravenous fluid containing sodium chloride or lactated Ringer’s solution given as boluses or continuous maintenance infusions not specified as medications or piggybacks. Admission weight was abstracted from the earliest weight recorded in an approach similar to prior studies, measured on a bed scale either in the Emergency Department or on arrival to the ICU [21]. Missing laboratory and physiologic values for the time periods of interest were assumed to be normal.

### Statistical analysis

Descriptive statistics were computed for ICU and patient characteristics. Covariates were plotted to explore distributions and suitable categorizations for statistical modeling. Differences in baseline characteristics by diuretic use were explored in bivariate analyses using Student’s *t* tests, Wilcoxon rank-sum tests, and chi-square tests as appropriate. To confirm clinical eligibility for diuretic use across groups, we also assessed for differences in physiological variables (shock, AKI, CVP, electrolyte derangements) used in the FACTT conservative strategy protocol for which clinicians were instructed against or permitted exceptions to diuretic use [11].

We used several approaches to evaluate the associations between fluid balance, diuretic use, and hospital mortality. Cumulative ICU fluid balance over time, hospital mortality, and liberation from mechanical ventilation were plotted over time by diuretic use at ARDS hours 48 to 72. Volume overload at 48 h was a clinically meaningful measure of fluid balance and treated as a confounding factor associated with both diuretic use and hospital mortality. Similarly, the use of minimal crystalloid fluid is likely to be associated with early diuretic use and was considered a potential confounder.

Multivariable logistic regression was performed to evaluate the associations between early diuretic use and hospital mortality controlling for confounders identified a priori. In the final multivariable model, we included age, SOFA score at ARDS onset, heart failure, trauma status, volume overload at 48 h after ARDS, and shock at 48 to 72 h. Crystalloid use and shock were not included, due to high collinearity with other exposures.

Sensitivity analyses were performed by assessing for effect-modification with each confounder. We also repeated the analyses stratifying by potentially influential subgroups—sepsis, ICU type, history of heart failure, and FACTT protocol instructions—to explore the robustness of results. Analyses were conducted using SAS version 9.4 (Cary, NC).

## Results

**Fig. 1:**
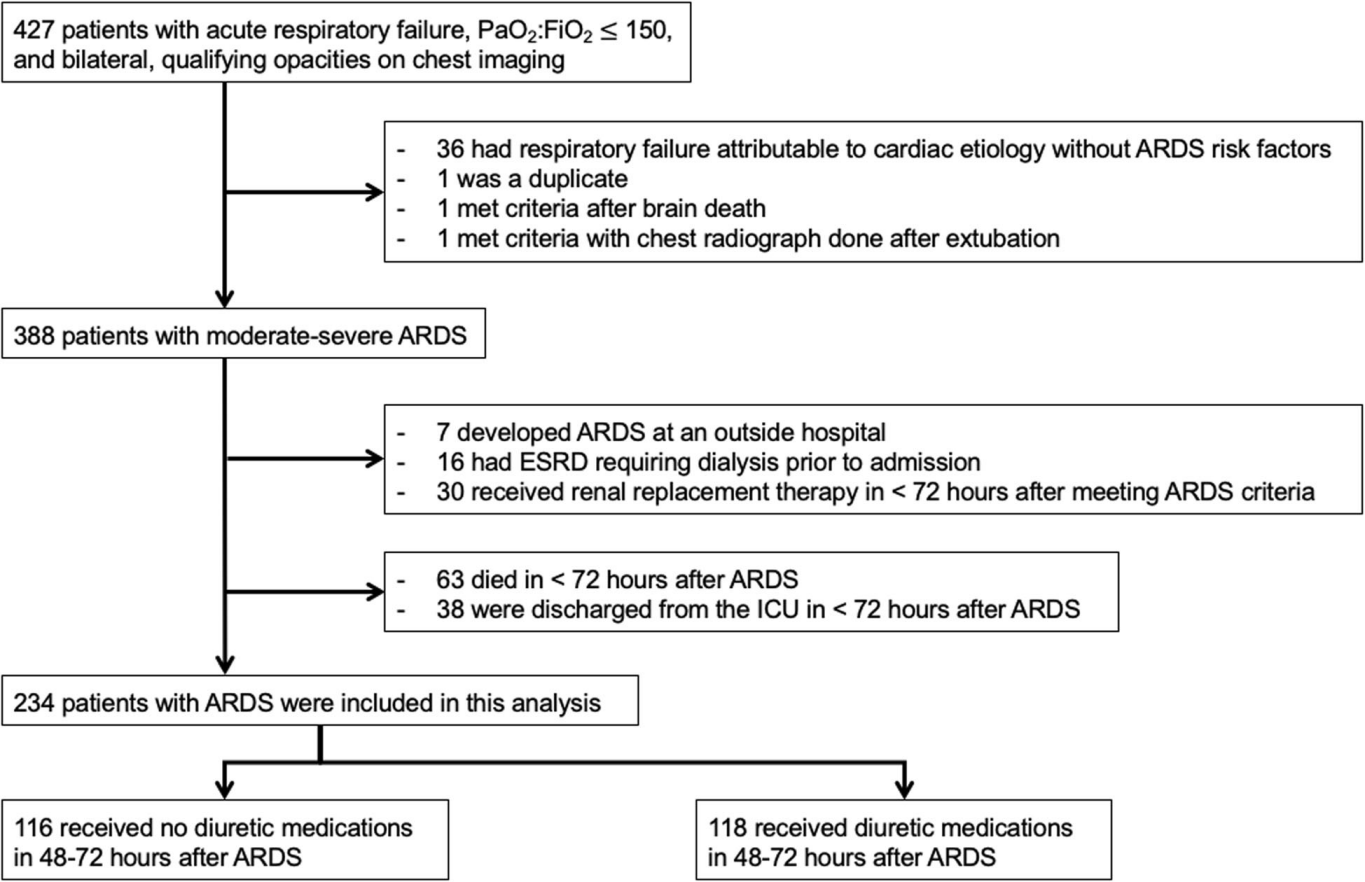
Study flow chart. ARDS = acute respiratory distress syndrome; ESRD = End-stage renal disease

Four hundred and twenty-seven patients were screened for inclusion with qualifying respiratory failure and chest imaging during the study period (Fig. 1). Of these, 388 patients met moderate-severe ARDS criteria. From these eligible patients, 234 were alive in the ICU at 72 h after meeting ARDS criteria, did not receive early renal replacement therapy, and were included in this analysis. Most patients in this sample were in the hospital 1 day or less before developing ARDS, and for most patients, mechanical ventilation was initiated on the same day that they met ARDS criteria (Table 1). Between 48 and 72 h after ARDS, 50% of the patients received a dose of diuretic medication. Of the patients who received diuretics, 108 (93%) received furosemide, six (5%) of the remaining patients received bumetanide and two (2%) received only diuretics other than loop diuretics. The median cumulative furosemide dose over 24 h was 40 mg (interquartile range: [20–80 mg]) (Supplemental Table 1).

**Table 1:** Patient characteristics by diuretic exposure in 48–72 h after meeting acute respiratory distress syndrome criteria

Patient characteristics^**a**^ (units)	No diuretic in 48–72 h after ARDS	Received diuretic in 48–72 h after ARDS	***P*** value
No. of patients	118	116	
**Demographics:**			
Gender (female)	35 (30%)	42 (36%)	0.29
Race (non-white)	26 (23%)	15 (13%)	0.07
Age (years)	56 (17)	57 (15)	0.59
Admission weight (kg)	82.5 [66.1-104.4]	82.5 [70.1-102.4]	0.63
**Comorbidities:**			
Congestive heart failure	**38 (32%)**	**61 (53%)**	0.002
Chronic lung disease	38 (32%)	37 (32%)	0.96
Cirrhosis	5 (4%)	4 (3%)	0.75
**ARDS risk factors:**			
Sepsis	57 (48%)	55 (47%)	0.89
Trauma	**46 (39%)**	**26 (22%)**	0.006
**ICU admission from operating room**	17 (14%)	21 (18%)	0.44
**ICU type:**	-	-	0.0003^b^
Medical ICU	**37 (31%)**	**18 (16%)**	
Cardiothoracic ICU	**12 (10%)**	**33 (28%)**	
Trauma-surgical ICU	**38 (32%)**	**27 (23%)**	
Other ICU	**31 (26%)**	**38 (33%)**	
**Prior to ARDS:**			
Hospital length of stay (days)	1 [0–4]	1 [0–3.5]	0.44
Mechanical ventilation (days)	0 [0–1]	0 [0–1]	0.15
SOFA score, day 0	10.3 (2.9)	10.5 (2.9)	0.64
**During ARDS hours 0–48:**			
Shock	85 (78%)	88 (76%)	0.51
Acute Kidney Injury	22 (19%)	15 (13%)	0.23
Lowest PaO2:FiO2 ≤ 150	118 (100%)	116 (100%)	
Any CVP measured	**24 (20%)**	**44 (38%)**	0.0031
Any transfusion	32 (27%)	31 (27%)	0.95
**Volume overload at 48 h**	30 (25%)	19 (16%)	0.09
**During ARDS hours 48–72:**			
Shock	55 (47%)	48 (41%)	0.42
Acute Kidney Injury	19 (16%)	12 (10%)	0.19
Lowest PaO2:FiO2 ≤ 150	11 (9%)	16 (14%)	0.28
Any CVP measured	**10 (8%)**	**32 (28%)**	0.0001
Any transfusion	13 (11%)	6 (5%)	0.1
Weight measured	**68 (58%)**	**85 (73%)**	0.012
Total furosemide dose^c^ (mg/24 h)	–	40 [20-80]	–
**Outcomes**			
RRT in first 7 days after ARDS	5 (4%)	2 (2%)	0.26
Duration of MV, all (days)	**7 [3–13]**	**5 [3–9]**	0.034
Duration of MV, survivors (days)	5 [3–13]	5 [3–8.5]	0.13
Discharge home self-care	37 (31%)	47 (41%)	0.15
Died in hospital	**30 (25%)**	**16 (14%)**	0.026

Acute kidney injury is defined as serum creatinine ≥2 mg/dL or urine output <500 mL/day

Shock is defined as vasopressor use or mean arterial pressure <60 mmHg for two measurements

*Abbreviations*: *ARDS* acute respiratory distress syndrome, *ICU* intensive care unit; *SOFA* Sequential Organ Failure Assessment, *CVP* central venous pressure, *RRT* renal replacement therapy

^a^Data shown as *n* (%), mean (standard deviation) or median [inter-quartile range] if not normally distributed

^b^*p* value for ICU type represents chi-square test for independence across ICU types

^c^Of patients who received diuretics in 48–72 h after ARDS, 108 (93%) received furosemide

Demographics were similar across groups with and without diuretic. The patients receiving diuretics had a higher prevalence of chronic congestive heart failure, and they were more often in the cardiothoracic ICU and less often in medical ICUs. Fewer patients receiving diuretics had trauma as an ARDS risk factor, but both groups had similar proportions of patients with sepsis. At the onset of ARDS, both had similar SOFA scores and ICU fluid balances. By hour 72 of ARDS, the groups also had overall similarly high rates of shock (44%, 95% CI: [38%, 51%]), low rates of AKI (13%, [9%, 18%]), and low prevalence of persistently severe ARDS (12%, [8%, 16%]). During ARDS hours 48 to 72, the group that received a diuretic more often had a CVP measurement recorded, but the proportion in both groups was low (8% vs 28%, *p* = 0.0001).

### Fluid balance

**Fig. 2:**
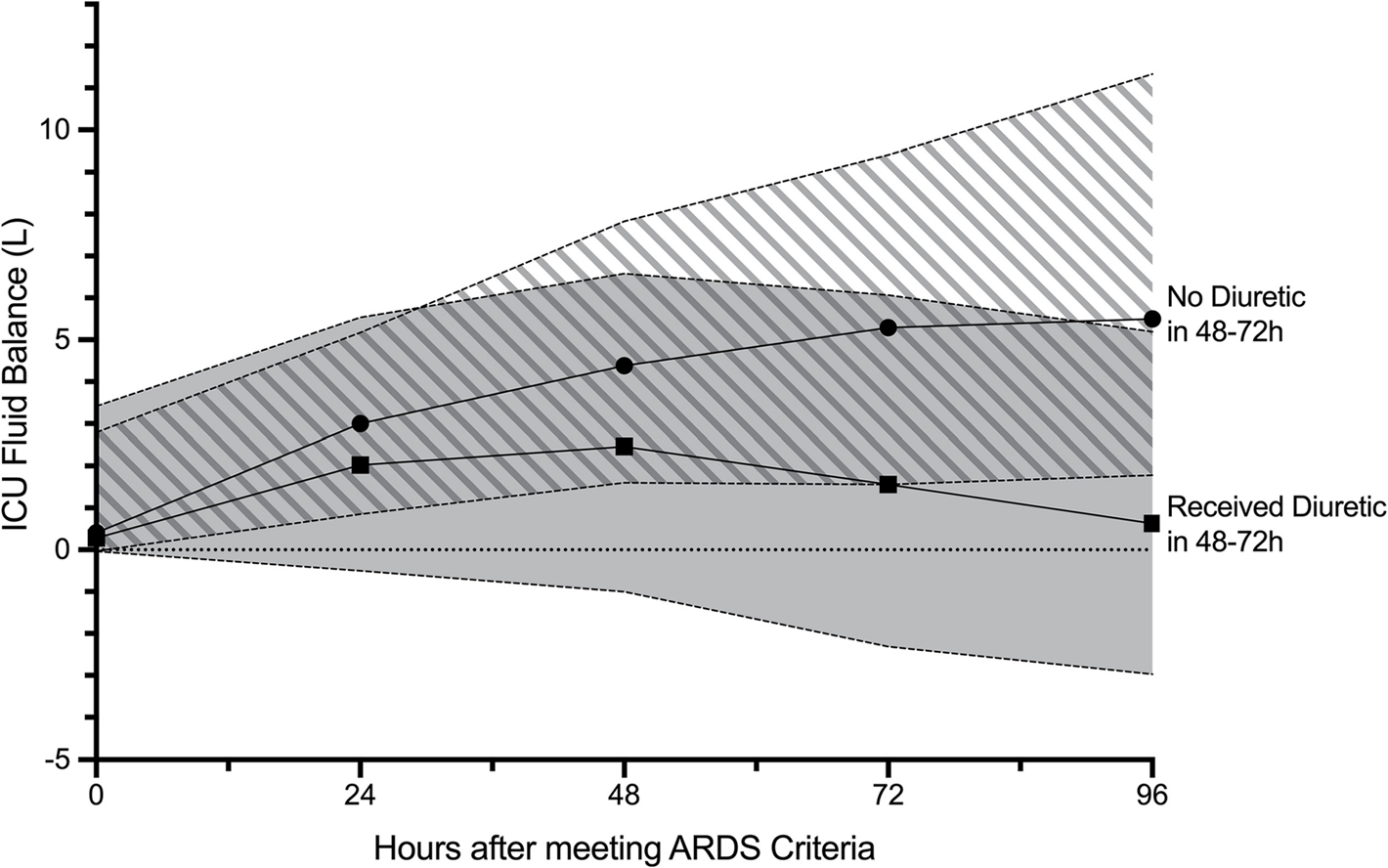
Distribution of cumulative ICU fluid balances over time, comparing subjects who received a diuretic in hours 48–72 after meeting ARDS criteria (“Received diuretic”, squares) with those who did not (“No diuretic”, circles). Data points represent median values for each group, with associated bands representing inter-quartile ranges. For hours 0–72, *n* = 234. For hour 96, *n* = 225

Both groups had similar net ICU fluid balances prior to ARDS, with median volumes close to zero (Table 2). In the next 48 h, however, those who did not receive diuretics were given approximately twice as much crystalloid fluid on average as those who did (median [inter-quartile range]: 2.4 L [1.2–5.0] vs 1.2 L [0.2–2.8], respectively; *p* < 0.001). The majority of fluid administered overall was not crystalloid but medication fluids or nutrition. At 48 h after ARDS, the group that received diuretics had a remarkably lower net fluid balance (Fig. 2). During hours 48 to 72, most patients received less than 500 mL of crystalloid, but at 72 h, the difference in median fluid balances had increased to 3.7 L (Supplemental Table 1). At 72 h, the proportion of patients that met volume overload criteria was twice as high in the group that did not receive diuretics compared with those that did (34%, 95%CI: [25%, 43%] vs 16% [10%, 24%]; *p* = 0.02).

**Table 2:** Fluid Balance by diuretic exposure in 48–72 h after meeting acute respiratory distress syndrome criteria

Patient characteristics^**a**^ (units)	No diuretic in 48–72 h after ARDS	Received diuretic in 48–72 h after ARDS	***P*** value
**Pre-ARDS ICU fluid balance** (total L)	0.4 [0–2.8]	0.3 [0–3.4]	0.89
**0–48 h after ARDS:**			
Daily fluid balance (L/24 h)	**1.5 [0.4–2.7]**	**0.5 [−0.7–1.7]**	<0.0001
Crystalloid intake (L/24 h)	**1.2 [0.6–2.5]**	**0.6 [0.1–1.4]**	<0.0001
Medication fluid intake (L/24 h)	1.1 [0.8–1.7]	1.1 [0.7–1.8]	0.92
Nutrition intake (L/24 h)	0.5 [0.1–1.1]	0.5 [0.2–1.0]	0.86
Free water intake (L/24 h)	0.1 [0–0.2]	0.1 [0–0.2]	0.39
Transfusion intake (L/24 h)	0 [0–0.1]	0 [0–0.1]	0.84
Total fluid output (L/24 h)	**2.2 [1.4–3.4]**	**2.7 [1.9–3.4]**	0.042
Net ICU fluid balance at 48 h (total L)	**4.4 [1.6–7.8]**	**2.5 [−1.0–6.6]**	0.0047
**48–72 h after ARDS:**			
Daily fluid balance (L/24 h)	**0.6 [−0.3–1.7]**	**−0.9 [−2.1–0.2]**	<0.0001
Crystalloid intake (L/24 h)	**0.3 [0–1.0]**	**0 [0–0.3]**	0.0007
Less than 500 mL in 24 h	**69 (59%)**	**84 (72%)**	0.03
Free water intake (L/24 h)	**0 [0–0.1]**	**0 [0–0.2]**	0.17
Medication fluid intake (L/24 h)	0.9 [0.5–1.5]	1 [0.5–1.6]	0.6
Nutrition intake (L/24 h)	0.9 [0.2–1.5]	1.1 [0.4–1.6]	0.42
Free water intake (L/24 h)	0 [0–0.1]	0 [0–0.2]	0.17
Transfusion intake (L/24 h)	0 [0–0]	0 [0–0]	0.05
Total fluid output (L/24 h)	**2.2 [1.6–2.9]**	**3.3 [2.4–4.5]**	<.0001
**Net ICU fluid balance at 72 h** (Total L)	**5.3 [1.6–9.4]**	**1.6[−2.3–6.1]**	<0.0001

Volume overload is defined as a net ICU fluid balance (in L) equivalent to more than 10% of admission body weight (in kg)

Duration of MV refers to the duration in days of mechanical ventilation episode that includes ARDS onset

*Abbreviations*: *ARDS* acute respiratory distress syndrome, *ICU* intensive care unit

^a^Data shown as *n* (%), mean (standard deviation), or median [inter-quartile range] if not normally distributed

### Comparing diuretic use to clinical trial protocol

The FACTT study protocol provided instructions for holding diuretics in renal failure and within 12 h of shock. During 48–72 h after ARDS in this cohort, the overall prevalence of acute kidney injury was low (13%, 19/234) with no difference between groups (Supplemental Table 2). Shock was common in this population overall but was not associated with diuretic use (47% in the no diuretic group vs 41% in the diuretic group; *p* = 0.42). Protocol instructions also included not giving diuretics to some patients with a CVP ≤ 8 cm H_2_O, and electrolyte derangements were monitored as an adverse outcome. In our cohort, both of these circumstances were very rare. In sum, these clinical factors could represent criteria for not giving diuretics per the FACTT protocol, but we found they were not associated with diuretic use in routine practice (51% vs 50%, respectively; *p* = 0.90).

### Outcomes

**Fig. 3:**
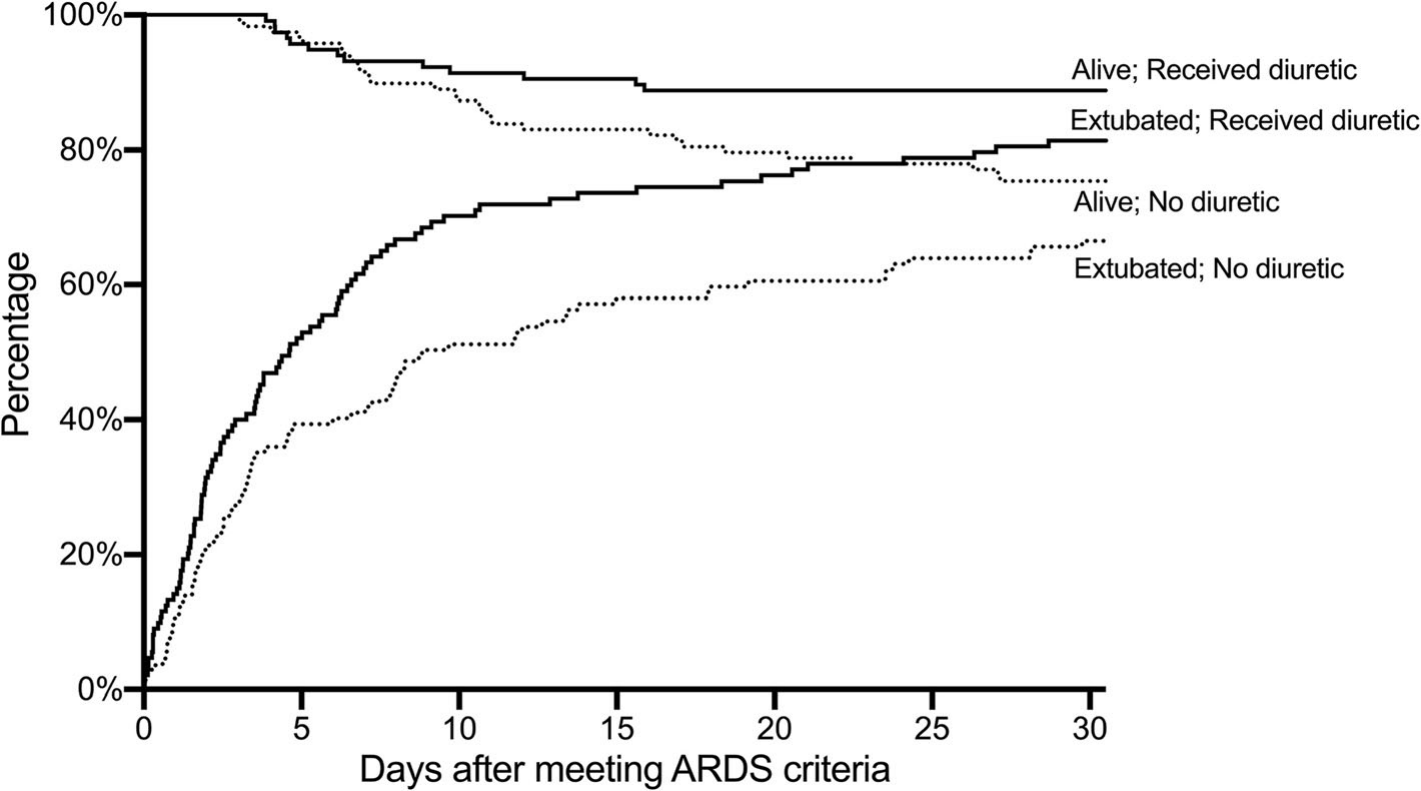
Proportion of patients surviving to hospital discharge (“Alive”) and proportion breathing without mechanical ventilation (“Extubated”), comparing subjects who received a diuretic during hours 48–72 after meeting ARDS criteria (solid line, “Received diuretic”) with those who did not (dotted line “No diuretic”). Time points for breathing without mechanical ventilation, represent the times of final extubation from mechanical ventilation during the study hospitalization among patients who survived to discharge. Overall, 4 patients died more than 30 days after meeting ARDS criteria, and 16 patients survived to discharge without mechanical ventilation but were extubated more than 30 days after meeting ARDS criteria

Forty-six patients (20%; 95% CI: [15%, 25%]) died in the hospital with a higher rate in the group that did not receive early diuretic medications (25% vs 14%; *p* = 0.03) (Fig. 3). In bivariate analyses, age, SOFA score at onset of ARDS, AKI, less than 500 mL of crystalloid fluid given, and diuretic use during hours 48 to 72 each were associated with hospital mortality (Supplemental Table 3).

A multivariable logistic regression model with confounders chosen a priori was constructed to explore the association between early diuretic use and hospital mortality. In the final model, diuretic use at 48 to 72 h was independently associated with hospital mortality by an adjusted odds ratio of 0.46 (95%CI: [0.22, 0.96]), even after controlling for age, SOFA score at onset of ARDS, history of congestive heart failure, trauma status, and volume overload at 48 h (Table 3). Of note, volume overload at 48 h was not independently associated with mortality in the multivariable model but has collinearity with diuretic use that did not reach statistical significance.

**Table 3:** Multivariable model for hospital mortality

Patient characteristics	Adjusted odds ratio (95%CI)
Diuretics, 48–72 h after ARDS	**0.46 (0.22, 0.96)**
Age, categorized	**1.83 (1.14, 2.95)**
Congestive heart failure	0.59 (0.26, 1.34)
Trauma as ARDS risk factor	0.52 (0.22, 1.24)
SOFA score, at ARDS onset, categorized	**6.35 (1.91, 21.13)**
Volume overload at 48 h	1.65 (0.74, 3.71)

Age at admission was categorized into groups of <55, 55–70, 70–80, >80 years old.

SOFA score was categorized into groups of ≤8, >8 and ≤ 16, and >16 for a linear association with mortality

Volume overload is defined as a net ICU fluid balance (in L) equivalent to more than 10% of admission body weight (in kg)

*Abbreviations*: *ARDS* acute respiratory distress syndrome, *SOFA* Sequential Organ Failure Assessment Score

For secondary outcomes, the differences by diuretic-use group in the duration of mechanical ventilation among survivors and in proportion discharged to home self-care were not statistically significant.

### Sensitivity analyses

We conducted sensitivity analyses for the final model by sequentially excluding pre-specified sub-groups by patient factors and ICU types to confirm the association between diuretic use and mortality. By this method, we found no statistically significant difference in estimates of the association between diuretic use and mortality. Notably, the unadjusted association between diuretic use and mortality among patients without heart failure was of lesser magnitude (*n* = 135; OR 0.90, 95%CI [0.37,2.14]) than within the subgroup with heart failure (*n* = 99; 0.19 [0.06, 0.55]).

When the analysis was limited only to those who remained alive and in the ICU after 7 days (*n* = 160) to exclude both the highest and lowest acuity patients, the association between diuretic use and outcome in the multivariable model was unchanged.

## Discussion

This investigation used retrospective data to assess whether early conservative fluid management with diuretics for ARDS is associated with improved outcomes and whether this relationship is independent of other patient and ICU factors. Our analysis demonstrates three important findings.

First, one-half of these ARDS patients received diuretic medications early, in 48 to 72 h after ARDS. We found this diuretic use was not associated with the protocol instructions used in FACTT. Second, volume overload was common and present early, with crystalloid fluids comprising a relatively small proportion of daily fluid intake. Third, early diuretic use in ARDS is associated with better outcomes, as the cohort that received diuretics had lower hospital mortality even after controlling for confounding clinical factors.

### Comparison with previous studies

Volume overload is associated with organ failure and is recognized as an independent predictor of poor outcomes [19, 21, 23–25]. Increasing evidence demonstrates that restrictive fluid strategies including diuresis may be associated with better outcomes, particularly in critical illness and ARDS [10, 17, 26–29]. To our knowledge, this is the first study to characterize fluid balance and diuretic use in usual care for ARDS patients since Wiedemann and colleagues published the landmark trial, FACTT, in 2006 [11]. Of patients with ARDS that were screened for FACTT, 91.3% (10,511/11,512) were excluded. In contrast, to reflect usual care, we excluded only those already on dialysis and those who were ineligible for our exposure of interest.

FACTT compared a conservative and a liberal fluid management protocol in ARDS patients, with the conservative arm receiving more diuretic medications and less crystalloid. The trial demonstrated a benefit to the conservative protocol in lung function, duration of mechanical ventilation, and ICU stay. Our findings also suggest benefit from early conservative fluid management. Notably, FACTT did not find a mortality difference attributable to the fluid management strategy, where our analysis found a strong association. This difference may be explained by the FACTT protocol instructions that provided multiple criteria for not giving diuretics in the conservative arm, where all patients in our exposed group received diuretics.

On day 2 after enrollment in FACTT, 60% of subjects in the conservative arm of the trial received furosemide, while 29% did in the liberal arm. In our study population, 50% overall received a dose of diuretic. The clinical trial achieved a 4.7 L separation in the mean net fluid balance between each arm, while the two groups in our cohort had a difference of 3.7 L in median ICU fluid balance at a comparable time point of 72 h from ARDS onset.

In contrast to the study population of FACTT, we only included patients with moderate-severe ARDS defined by a PaO_2_:FiO_2_ ratio of less than 150, where FACTT included all ARDS patients with a ratio less than 300. Our cohort was similar in age to the population enrolled in FACTT (mean ages 55 and 50 years, respectively) and observed mortality (20 and 27%). While all FACTT care protocols required CVP or PAOP values, these measurements were rarely used in our cohort, with only 18% having any CVP documented during ARDS hours 48 to 72. Finally, FACTT excluded many ARDS patients such as those with chronic lung disease or severe cirrhosis, and its protocol withheld diuretics from those with shock, with a normal CVP, or renal failure. We found no difference in the management of patients by these factors, demonstrating that they are not clinical exclusions to diuretic use in usual care. The benefits of conservative fluid management and the use of diuretics may be generalizable to these populations, which would be consistent with clinician surveys and observational studies [30–32].

Prior studies have demonstrated that most fluids administered in critical care are given with medications rather than as bolus or maintenance crystalloid [33, 34]. Our study corroborates these findings that crystalloid used for resuscitation is typically a less significant contributor to net fluid balance than diuretic use and incidental fluids in the ARDS population [29, 35–38].

Gaps in implementation of evidence from randomized controlled trials into routine care for ARDS have been demonstrated previously by the inconsistent use of low tidal volume ventilation or prone positioning [3, 39–41]. Early attention to fluids and diuresis may represent another implementation gap from under-recognition of ARDS or from missed opportunities to provide early conservative fluid management in standard practice.

### Study limitations

There are several potential limitations to this study. Confounding by indication poses the largest threat to the validity of this analysis. We do not have direct measures of clinical decision-making for diuretic use in usual care. CVP or PAOP criteria were used to guide management in both the FACTT and FACTT Lite study protocols, but these measurements were rarely available in our ARDS population [11, 16]. Fluid balance may affect diuretic use and outcomes, so we conducted sensitivity analyses and included volume overload as a confounder in the multivariable model. Variability in opinion exists among providers on the best methods for volume status assessments and fluid management decisions, suggesting a likely source of provider-level variability [31, 42]. Nevertheless, the groups of patients may have had other unmeasured confounders responsible for the difference in outcomes.

We identified a cohort with moderate-severe ARDS to reflect current clinical practice and ongoing clinical trials, but heterogeneity among ARDS patients certainly exists and effect modification may be present in influential sub-groups [43–45]. Sensitivity analyses did not identify any groups with significantly different benefits or harm in this sample, but this study was underpowered to detect meaningful associations within sub-groups. A strong association exists between diuretic use and mortality in the sub-group enriched for congestive heart failure, while considerable uncertainty exists in the estimate for the group without heart failure. We have no direct measurement of left atrial pressures in this observational study, but data collected in FACTT demonstrated no association between elevated left atrial pressure and outcomes [46, 47]. These findings are a further demonstration of the abundance of comorbidities like heart failure in the ARDS population, and emerging research may better identify distinct disease processes combined into this cohort.

Our exposure of interest was receiving diuretics during a 24-h sample time period, but one dose is not expected to have a direct causal effect on mortality. Rather, this measure serves as a marker for early attention to conservative fluid management and diuresis. Receiving diuretics during this time is highly correlated with additional use across the ICU course. Of those patients who received a diuretic in 48 to 72 h after ARDS, 74% (86/116) also received a dose during the following day (72 to 96 h), compared with only 25% (29/118) of the other group. We found that the use of this dichotomous variable at an early time point effectively captures an eligible cohort and simplifies the assessment and differentiation of groups while minimizing the multiple comparisons of longitudinal analyses.

Fluid balance was an important covariate in this retrospective study, but it was subject to multiple sources of bias. The assessments of fluid intake and output were limited to data that were documented in the electronic medical record. As a consequence, patients who had an output that was challenging to quantify, like blood or gastrointestinal losses, were at risk for measurement bias, as were those who visited the operating rooms, where fluid balance documentation may be different from standard practice in the ICU. Additionally, we defined volume overload as a cumulative ICU fluid balance greater than 10% of the earliest body weight recorded during the hospital encounter, based on prior published research from our institution [21]. Some patient weights, however, may have been measured inaccurately or after initial fluid resuscitation, which could have led to misclassification of volume overload status.

Finally, this study was conducted at two hospitals in the same city, which limits the generalizability due to local clinician practice patterns and ICU populations with a relatively high proportion of trauma as an ARDS risk factor.

## Conclusions

This study is an important contribution to the literature on fluid management in ARDS. We demonstrate that the use of diuretic medications in this cohort is a highly variable practice that is associated with outcomes. Our analysis provides insight and support for active fluid stewardship in most ARDS patients. Increasing the prevention, recognition, and early treatment of volume overload in ARDS patients may be key implementation opportunities to improve outcomes for patients with ARDS. These observations can also inform an interventional study, to determine the impact of reducing variability for ARDS care and standardizing fluid management with active diuresis as a target.

Among patients with ARDS, volume overload is common and present early. Most patients accumulate a positive net fluid balance after meeting ARDS criteria, driven by fluid intake from sources other than crystalloid. Early diuretic use after meeting ARDS criteria is independently associated with lower hospital mortality.

## Supplementary information

**Additional file 1: Supplemental Table 1.** Cumulative ICU fluid balance and volume overload prevalence over time by diuretic exposure in 48-72 hours after meeting ARDS criteria. **Supplemental Table 2.** Prevalence of potential exceptions to diuretic use by diuretic exposure in 48-72 hours after meeting ARDS criteria. **Supplemental Table 3.** Univariate associations with hospital mortality.

## Data Availability

The datasets used and/or analyzed during the current study are available from the corresponding author on reasonable request.

## Abbreviations

AKI: Acute kidney injury
ARDS: Acute respiratory distress syndrome
CVP: Central venous pressure
FACTT: Fluid and Catheters Treatment Trial
FiO_2_: Fraction of inspired oxygen
ICU: Intensive care unit
PAOP: Pulmonary artery occlusion pressure
PaO_2_: Partial pressure of oxygen in arterial blood
SOFA: Sequential Organ Failure Assessment
