# Supplementary material for: Fluid management in ARDS: an evaluation of current practice and the association between early diuretic use and hospital mortality
Source: J Intensive Care. 2020 Oct 12;8:78. doi: 10.1186/s40560-020-00496-7 (PMC7549083; doi:10.1186/s40560-020-00496-7)
Supplement: Supplementary file 1 — Additional file 1: Supplemental Table 1. Cumulative ICU fluid balance and volume overload prevalence over time by diuretic exposure in 48-72 hours after meeting ARDS criteria. Supplemental Table 2. Prevalence of potential exceptions to diuretic use by diuretic exposure in 48-72 hours after meeting ARDS criteria. Supplemental Table 3. Univariate associations with hospital mortality. [file 40560_2020_496_MOESM1_ESM.docx]

Supplemental Table 1: Cumulative ICU fluid balance and volume overload prevalence over time by diuretic exposure in 48-72 hours after meeting ARDS criteria

| **Time after ARDS criteria** | **No diuretic in 48-72 hours after ARDS**  (N=118; N=112 at 96h) | | | **Received diuretic in 48-72 hours after ARDS**  (N=116; N=113 at 96h) | | |
| --- | --- | --- | --- | --- | --- | --- |
|  | *Net ICU fluid balance (total L)^a^* | *Volume overload* ^b^ | *Furosemide dose*  *(total mg/24h)^c^* | *Net ICU fluid balance*  *(total L)* | *Volume overload* | *Furosemide*  *dose*  *(total mg/24h)^d^* |
| at 0h | 0.4 [0-2.8] | 12 (10%) |  | 0.3 [0-3.4] | 12 (10%) |  |
| at 24h | 3.0 [0.9-5.2] | 20 (17%) | 0 [0-0] | 2.0 [-0.5-5.5] | 16 (14%) | 0 [0-21] |
| at 48h | 4.4 [1.6-7.8] | 30 (25%) | 0 [0-0] | 2.5 [-1.0-6.6] | 19 (16%) | 20 [0-60] |
| at 72h | 5.3 [1.6-9.4] | 40 (34%) | 0 [0-0] | 1.6 [-2.3-6.1] | 19 (16%) | 40 [20-80] |
| at 96h | 5.5 [1.8-11.3] | 39 (35%) | 0 [0-0] | 0.6 [-3.0-5.2] | 18 (16%) | 30 [0-80] |

*Definition of abbreviation:* ARDS = Acute Respiratory Distress Syndrome

^a^ Net ICU fluid balance presented as Median [Inter-quartile Range].

^b^ Volume overload presented as n(%).

*^c^* Furosemide dose is reported for time period 24 hours prior to timepoints and presented as Median [Inter-quartile Range]. For 0-24 hours after ARDS, 29 (25%) patients received furosemide, 22 (19%) did during 24-48 hours, 0 (0%) did during 48-72 hours, and 29 (25%) did from 72-96 hours.

^d^ In the diuretic group, for 0-24 hours after ARDS, 38 (33%) patients received furosemide, 64 (55%) did during 24-48 hours, 108 (93%) did during 48-72 hours, and 79 (68%) did from 72-96 hours.

Volume overload defined as a net ICU fluid balance (in L) equivalent to more than 10% of admission body weight (in kg).

Supplemental Table 2. Prevalence of potential exceptions to diuretic use by diuretic exposure in 48-72 hours after meeting ARDS criteria

| **Patient characteristics at 48-72h after ARDS^a^** | **No diuretic in 48-72 hours after ARDS** (n=118) | **Received diuretic in 48-72 hours after ARDS** (n=116) | **P-value** |
| --- | --- | --- | --- |
| Shock | 55 (47%) | 48 (41%) | 0.42 |
| Normal CVP | 3 (3%) | 4 (3%) | 0.68 |
| Acute Kidney Injury | 19 (16%) | 12 (10%) | 0.72 |
| Electrolyte Derangement | 1 (1%) | 3 (3%) | 0.37 |
| Any of the above criteria | 60 (51%) | 58 (50%) | 0.90 |

*Definitions of abbreviations:* ARDS = Acute Respiratory Distress Syndrome; AKI = Acute Kidney Injury; CVP = Central Venous Pressure

^a^Data shown as n(%).

Shock defined as vasopressor use or mean arterial pressure < 60 mmHg for two measurements.

Normal CVP refers to a recorded Central Venous Pressure < 9 mmHg.

AKI defined as serum creatinine ≥ 2 mg/dL or urine output < 500 mL/day.

Electrolyte derangements include any of the following: Sodium >150 mEq/L, Potassium < 3 mEq/L, or Bicarbonate > 40 mEq/L.

Supplemental Table 3. Univariate associations with hospital mortality

| **Patient characteristics** | **Odds Ratio *(95% CI)*** |
| --- | --- |
| Age, categorized | **1.69 (1.14, 2.48)** |
| Congestive heart failure | 1.06 (0.55, 2.03) |
| Sepsis as ARDS risk factor | 1.38 (0.72, 2.64) |
| Trauma as ARDS risk factor | 0.48 (0.22, 1.06) |
| ICU type (ref is Other) |  |
| Medical | 1.35 (0.59, 3.08) |
| Cardiothoracic | 0.78 (0.30, 2.02) |
| Trauma-Surgical | 0.51 (0.20, 1.29) |
| SOFA score, at ARDS onset, categorized | **6.46 (2.08, 20.04)** |
| Volume overload at 48h | 1.91 (0.92, 3.96) |
| Net ICU fluid balance < 0L at 48h | 0.45 (0.18, 1.13) |
| Vasopressor use, 48-72h after ARDS | 1.75 (0.91, 3.37) |
| Shock, 48-72h after ARDS | 1.87 (0.98, 3.59) |
| AKI, 48-72h after ARDS | **2.64 (1.16, 6.00)** |
| Less than 500mL crystalloid intake, 48-72h after ARDS | **0.50 (0.26, 0.99)** |
| Diuretic use, 48-72h after ARDS | **0.47 (0.24, 0.92)** |

*Definitions of abbreviations:* ARDS = Acute Respiratory Distress Syndrome; SOFA = Sequential Organ Failure Assessment Score; ICU = Intensive Care Unit; AKI = Acute Kidney Injury

Crystalloid refers to IV crystalloid fluids given as bolus or continuous infusion.

Age at admission was categorized into groups of <55, 55-70, 70-80, >80 years old. SOFA score was categorized into groups of ≤8, >8 and ≤16, and >16 for a linear association with mortality.

Volume overload defined as a net ICU fluid balance (in L) equivalent to more than 10% of admission body weight (in kg).

Shock defined as vasopressor use or mean arterial pressure < 60 mmHg for two measurements.

AKI defined as serum creatinine ≥ 2 mg/dL or urine output < 500 mL/day.
